# MPR-MOPSO-ASFS: A Stable Multi-Objective Feature Selection Algorithm for Metabolomics

**DOI:** 10.3390/metabo16080581

**Published:** 2026-08-17

**Authors:** Qing Ye, Zeng Deng, Xin Xie, Qiang Huang, Jigen Luo

**Affiliations:** 1 School of Intelligent Medicine and Information Engineering, Jiangxi University of Chinese Medicine, Nanchang 330004, China; 19900385@jxutcm.edu.cn (Q.Y.); dengzeng@jxutcm.edu.cn (Z.D.); xiexin8@jxutcm.edu.cn (X.X.); 20201041@jxutcm.edu.cn (Q.H.); 2 Jiangxi Provincial Engineering Research Center of Traditional Chinese Medicine Big Data and Artificial Intelligence, Nanchang 330004, China

**Keywords:** metabolomics, feature selection, multi-objective optimization, particle swarm optimization

## Abstract

**Background**: Metabolomics data is inherently characterized by high dimensionality and small sample size. Existing multi-objective particle swarm optimization (MOPSO)-based feature selection algorithms suffer from two critical limitations: they generally neglect the stability of selected feature subsets and exhibit poor adaptability when processing high-dimensional data, which restricts their practical application in biomedical research. **Methods**: To address these challenges, this paper proposes a novel MPR-MOPSO-ASFS algorithm. Specifically, we first construct a stability-driven bi-objective optimization model. Then, we integrate a Maximum Pattern Recognition (MPR) filter to achieve rapid dimensionality reduction of high-dimensional features. Finally, an improved Adaptive Sparsity Feature Selection mechanism is designed to simultaneously optimize the compactness, classification accuracy, and stability of the selected feature subsets. **Results**: Extensive experiments were conducted on three metabolomics datasets and five public high-dimensional small-sample datasets. The results demonstrate that the proposed MPR-MOPSO-ASFS algorithm outperforms mainstream algorithms including CMDPSOFS and MOEAD-FS in core evaluation metrics such as Pareto front quality and classification accuracy. Additionally, we clarify the optimal configuration of the algorithm’s core parameters and verify the collaborative effectiveness of its key design components. **Conclusions**: This study makes the first attempt to incorporate the harmonic mean of accuracy and stability into the multi-objective optimization framework. The two-stage combination of the proposed MPR strategy and MOPSO breaks through the performance bottleneck of traditional algorithms and significantly enhances their adaptability and robustness to high-dimensional small-sample data. The proposed algorithm provides an efficient new solution for feature selection in high-dimensional biomedical data and offers a technical reference for the application of swarm intelligence optimization algorithms in the biomedical field.

## 1. Introduction

As a core branch of systems biology, metabolomics provides key biomarker support for clinical research such as early cancer diagnosis and diabetes classification by analyzing the dynamic changes in small-molecule metabolites in organisms [1]. The efficient analysis and mining of metabolomics data rely on reliable optimization algorithms, among which Swarm Intelligence Algorithms are a widely adopted heuristic optimization paradigm. Inspired by the collective foraging and survival behaviors of natural biological populations, the core idea of swarm intelligence is to realize optimal solution search in high-dimensional space through information sharing and cooperative evolution among population individuals. Without depending on the gradient information of the objective function, such algorithms boast strong global search capability, favorable robustness, and easy implementation, and have been extensively applied to feature selection, biomarker identification, and other tasks in the biomedical field. However, the uniqueness of metabolomics data puts forward higher requirements for feature selection algorithms. On the one hand, the extreme dimensional imbalance of data easily leads algorithms to fall into local optima; however, the single improvement strategy of traditional MOPSO makes it difficult to balance convergence speed and population diversity [2]. On the other hand, metabolomics data has batch effects and individual differences, and the stability of feature subsets directly determines the reliability and clinical transformation value of biomarkers. Nevertheless, mainstream algorithms only take classification accuracy and feature number as core optimization objectives, completely ignoring the key demand of stability. Although some studies attempt to improve the performance of MOPSO through hybrid initialization or parameter adaptation, a collaborative optimization framework integrating accuracy, stability, and feature number has not been formed, which cannot meet the actual needs of metabolomics biomarker screening [3].

As a core preprocessing link in machine learning and data mining, feature selection aims to screen feature subsets with both correlation and complementarity from high-dimensional raw data, thereby eliminating the interference of redundant and noisy features, improving model generalization ability and reducing computational cost [4]. With the wide application of high-throughput detection technologies such as liquid chromatography–mass spectrometry (LC-MS) in the biomedical field, metabolomics data presents typical characteristics of high-dimensionality, small sample size, strong redundancy, and high noise. Traditional feature selection methods face the dual challenges of the curse of dimensionality and reliability verification of biomedical markers. At this time, the multi-objective optimization framework has become an ideal technical path for feature selection of high-dimensional metabolomics data by virtue of its ability to collaboratively balance conflicting objectives such as classification performance and feature number. Among them, Multi-Objective PSO (MOPSO) has become a mainstream framework for multi-objective optimization by introducing Pareto dominance relation, external archive set, and other mechanisms [2].

To address the stability-driven requirements of metabolomics data, this paper proposes a multi-objective feature selection algorithm, MPR-MOPSO-ASFS, that integrates Maximum Pattern Recognition (MPR) and Adaptive Sparsity Feature Selection (ASFS). The core objective of this study is to construct a bi-objective optimization model that minimizes the feature selection rate $f_{1}$ and the harmonic mean of accuracy and stability $f_{2}$. The proposed model addresses the deficiency of the existing MOPSO algorithm that ignores stability. The MPR strategy and ASFS mechanism are adopted to optimize the convergence–diversity balance of MOPSO and improve the adaptability of the algorithm to metabolomics data. The core contributions of this study are as follows: Firstly, the harmonic mean of accuracy and stability is incorporated into the multi-objective optimization framework of MOPSO for the first time. Secondly, the improved ASFS adaptive guide particle selection strategy is designed to balance the convergence and diversity of the algorithm by dynamically selecting guide particles, improve the optimization performance of multi-objective feature selection, and remedy the domain adaptation defects of general MOPSO. On multiple datasets covering different disease types, the MPR-MOPSO-ASFS algorithm is comprehensively compared with mainstream improved MOPSO algorithms such as CMDPSOFS, RFPSOFS, MOPSO-ASFS, and classic multi-objective algorithms such as NSGA-II. The superiority of the algorithm is systematically verified from four dimensions: Pareto front quality, classification accuracy, stability, and feature number [5].

The rest of this paper is organized as follows: Section 2 reviews the related research on metabolomics feature selection and multi-objective optimization algorithms. Section 3 elaborates on the problem definition and algorithm design of the MPR-MOPSO-ASFS algorithm. Section 4 verifies the algorithm performance through experiments and analyzes the results. Section 5 discusses the practical significance of the research findings, explains the internal mechanism of each core module’s effect, and confirms the reliability of the conclusions in combination with existing domain studies. Section 6 summarizes the main work and contributions of this study, points out the limitations of the current research, and prospects the future research directions.

## 2. Related Work

### 2.1. Stability and Accuracy Evaluation System in Metabolomics

Stability and accuracy are core indicators to measure the effect of metabolomics feature selection, and the scientificity of their evaluation system directly affects the reliability of research conclusions [6].

Stability evaluation indicators measure the consistency of feature subsets in different data subsets or multiple experiments, and are a key guarantee for the reliability of metabolomics biomarkers. Existing stability indicators can be divided into three categories: indicators based on set similarity, such as Jaccard similarity coefficient and Dice coefficient, which evaluate stability by calculating the overlap degree of feature subsets, with simple calculation but without considering feature importance; indicators based on correlation, such as Spearman correlation coefficient and Pearson correlation coefficient, which reflect stability by analyzing the consistency of feature ranking, suitable for ordered feature subsets; indicators based on statistical tests, such as Kuncheva’s S index, which quantify the significance of differences in feature subsets through hypothesis testing, with stronger robustness. In metabolomics research, Jaccard similarity coefficient and Kuncheva’s S index are the most widely used [7]. However, most existing studies use stability indicators alone without forming a collaborative evaluation system with accuracy and feature number, making it difficult to quantify the trade-off relationship between accuracy, stability, and feature number. In addition, existing stability indicators are sensitive to data batch effects and lack targeted correction mechanisms in multi-center and multi-batch metabolomics data, affecting the objectivity of evaluation results.

Accuracy evaluation indicators are mainly used to quantify the classification performance of feature subsets, and are divided into supervised learning and unsupervised learning indicators according to data types. In supervised learning scenarios of metabolomics, commonly used indicators include classification accuracy, F1-score, Area Under the Receiver Operating Characteristic Curve (AUC-ROC), etc. Among them, accuracy intuitively reflects the overall classification effect but is sensitive to imbalanced data; F1-score balances precision and recall, suitable for small-sample metabolomics data; and AUC-ROC effectively avoids the influence of data distribution deviation by depicting classification performance under different thresholds, and is a preferred indicator for metabolomics classification tasks. In unsupervised learning scenarios, common indicators such as the Silhouette Coefficient and the Davies–Bouldin Index evaluate the effectiveness of feature subsets by measuring intra-cluster compactness and inter-cluster separation, but have limited application in supervised learning tasks such as metabolomics disease diagnosis. Existing accuracy evaluation indicators are relatively mature, but lack a collaborative evaluation mechanism with stability, making it difficult to fully reflect the comprehensive effect of metabolomics feature selection.

### 2.2. Multi-Objective Optimization Algorithms in Feature Selection

Multi-objective optimization algorithms are core tools for multi-objective feature selection, and their development revolves around three core demands: convergence speed, front diversity, and domain adaptability. Mainstream algorithms can be divided into two categories: Evolutionary Algorithms (EAs) and Swarm Intelligence Algorithms.

Among Evolutionary Algorithms, NSGA-II, as a classic multi-objective optimization framework, efficiently generates uniformly distributed Pareto fronts through fast non-dominated sorting and crowding distance calculation. A Pareto front consists of non-dominated solutions—namely feature subsets that cannot improve one optimization objective without degrading at least one other objective. In metabolomics feature selection, these correspond to balanced optimal biomarker combinations that trade off between feature compactness and classification performance. NSGA-II has been widely used in the field of feature selection [8]. However, its selection pressure depends on population size, and it is prone to uneven front distribution in high-dimensional metabolomics data. Based on the decomposition idea, MOEA/D transforms multi-objective problems into multiple weighted single-objective subproblems, improves convergence speed through neighborhood collaboration, and is suitable for high-dimensional data, but it does not easily adapt fixed weight vectors to the complex feature distribution of metabolomics data [9]. Genetic Algorithm (GA), as the earliest Evolutionary Algorithm applied to feature selection, simulates biological evolution through crossover and mutation operators, but has the defects of slow convergence and easy prematurity, making it difficult to balance exploration and exploitation capabilities in metabolomics data [10].

Swarm Intelligence Algorithms have gradually become a research hotspot of multi-objective feature selection by virtue of their simple structure and efficient convergence, among which MOPSO and its variants are the most representative. Traditional MOPSO, based on the social collaboration and individual learning mechanism of particle swarms, stores non-dominated solutions through an external archive set, and shows faster convergence speed than NSGA-II in feature selection [8]. To solve the traditional MOPSO problems of insufficient population diversity and easy falling into local optima, researchers have proposed a series of improvement strategies: CMDPSOFS introduces crowding distance calculation, mutation mechanism and dominance relation optimization, enhances population diversity through uniform/non-uniform mutation operators, and improves search ability in high-dimensional data [11]; RFPSOFS innovatively constructs a feature elitism mechanism based on the occurrence frequency of features in the archive set, optimizes archive set quality and particle motion direction through feature ranking, and performs well on UCI high-dimensional datasets, but does not consider the interference of data noise on feature frequency [12]; and MOPSO-ASFS designs an adaptive penalty mechanism and a feature information-guided leader particle selection strategy based on the Penalty Boundary Intersection (PBI) decomposition strategy, which effectively enhances population selection pressure and improves the distribution uniformity of the Pareto front, but it does not easily adapt the fixed particle update rule to the small-sample characteristics of metabolomics data [13]. In addition, other Swarm Intelligence Algorithms such as Multi-Objective Grey Wolf Optimization and Multi-Objective Artificial Bee Colony have also been applied to feature selection, but they have problems such as complex parameter adjustment and poor adaptability to high-dimensional data, and have not been widely used in the field of metabolomics [10].

## 3. Materials and Methods

### 3.1. Dataset

To comprehensively explore the performance of multi-objective optimization algorithms after feature selection, this study selects three metabolomics datasets and five public high-dimensional small-sample datasets for research [14].

1. Shenfu Injection Experiment on the Treatment of Cardiogenic Shock (Mice, hereinafter referred to as EnSub)

This study constructed a rat model of mid-term cardiogenic shock based on ligation of the distal end of the left anterior descending coronary artery, and performed serum metabolomics analysis using Waters H-Class high-performance liquid chromatography coupled with Synapt G2-Si mass spectrometry. The experiment set a blank group (group C, n = 6), a model group (group M, n = 6), and 7 dose gradient administration groups (N1–N7, 0.1–20 mL·kg^−1^, n = 6 in each group), with a total of 54 samples. A total of 11,481 effective substances were detected by negative ion mode (*m*/*z* 100–1200). Exogenous substances were screened according to the frequency standard of ≥4 non-zero samples in the administration group, and the rest were determined as endogenous substances.

2. Study on Adenocarcinoma Lung Cancer (Human)

This study focuses on serum metabolomic biomarkers of lung adenocarcinoma. A total of 181 plasma samples were analyzed by gas chromatography–mass spectrometry (GC-MS), and the abundance data of 511 metabolites were obtained. The dataset and its detailed experimental design information are available in Metabolomics Workbench (Study ID: ST000385).

3. Asthma/Obesity Correlation Study (Mice)

This study aims to explore the effects of different dietary interventions on the arginine metabolic pathway in mouse lungs. A total of 80 lung tissue samples were analyzed by gas chromatography–mass spectrometry (GC-MS), and the abundance data of 614 metabolites were detected. The dataset is available in Metabolomics Workbench (Study ID: ST000419).

The remaining 5 datasets are all publicly available datasets, as shown in Table 1, and detailed information can be found in the Supplementary Materials.

### 3.2. MPR-MOPSO-ASFS

#### 3.2.1. Overall Algorithm Framework

**Figure 1:**
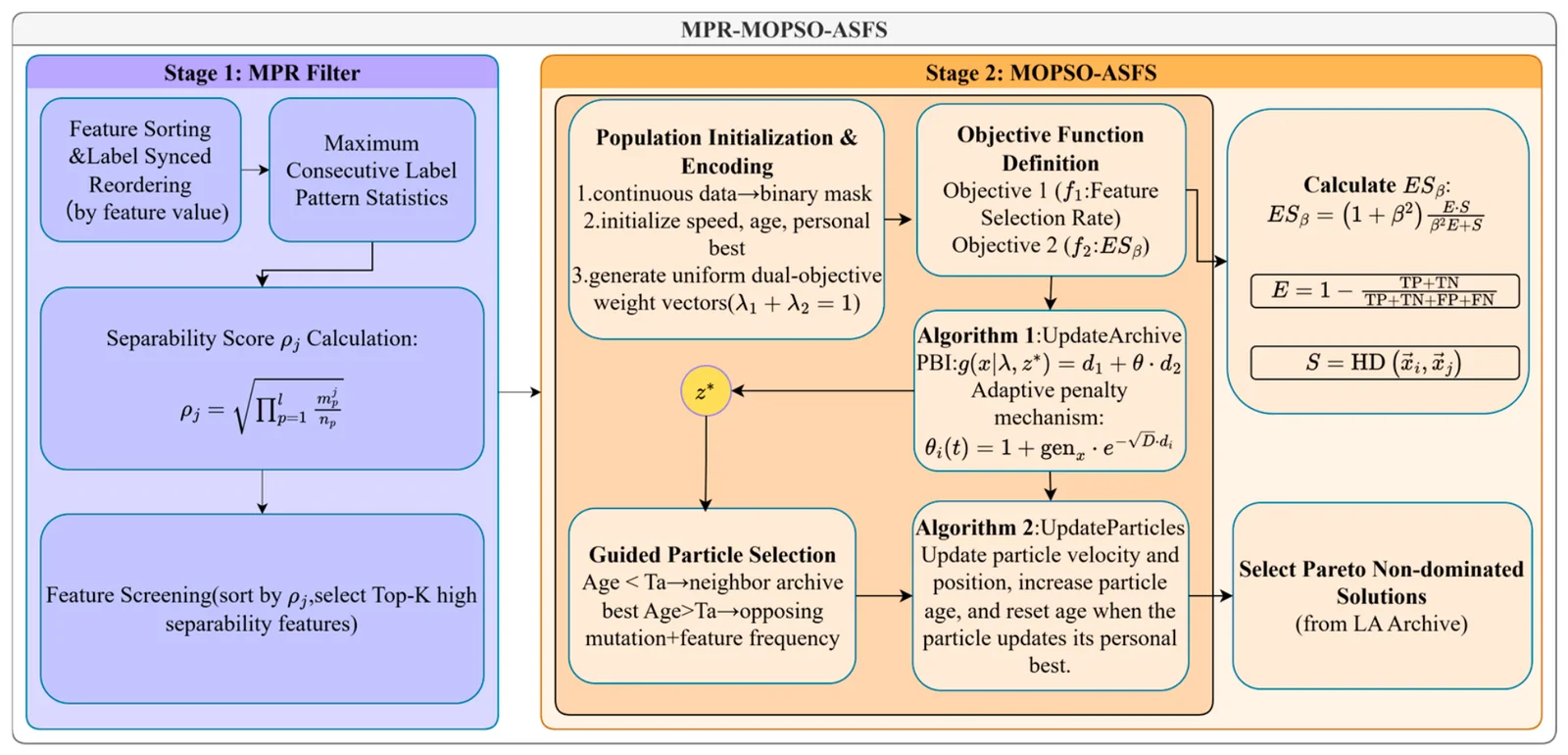
Overall framework of the MPR-MOPSO-ASFS algorithm.

Aiming at the feature selection problem of high-dimensional biomedical datasets, this paper proposes a two-stage hybrid feature selection algorithm: MPR-MOPSO-ASFS (Maximum Pattern Recognition-based Multi-Objective Particle Swarm Optimization with Adaptive Strategies for Feature Selection). The first stage uses the MPR filter to quickly evaluate and sort the original features, eliminate a large number of non-informative features, and compress the high-dimensional feature space into a small candidate feature set. The second stage runs the MOPSO-ASFS multi-objective optimization algorithm on the dimensionality-reduced feature subset. Through the adaptive penalty mechanism and adaptive guide particle selection strategy adapted to low dimensions, it searches for the Pareto optimal solution set between two conflicting objectives: feature selection rate and the joint indicator of accuracy and stability. The overall framework is shown in Figure 1.

#### 3.2.2. Maximum Pattern Recognition (MPR) Filter

In the feature selection task of high-dimensional biomedical datasets, the original feature space usually contains thousands to tens of thousands of features, while the number of samples is relatively small, which is a phenomenon known as the curse of dimensionality. Direct optimization search in high-dimensional space is not only computationally costly, but it also easily falls into local optimal solutions. Therefore, this study introduces the Maximum Pattern Recognition filter in the first stage to quickly eliminate a large number of non-informative features, compress the search space into a small candidate feature set (detailed parameter analysis is provided in Section 4.3.1), and lay a foundation for subsequent multi-objective optimization.

MPR is a frequency-based supervised filter ranker, and its core idea originates from statistical observations of feature separability. For a feature with good classification and discrimination ability, when samples are sorted according to the feature value, labels of the same category tend to appear continuously in the sorted sequence, forming a long continuous pattern. Maximum Pattern Recognition evaluates the separability strength of each feature by quantifying this pattern continuity. Compared with traditional frequency rankers, this method does not assume that the data obeys a specific distribution, can effectively identify non-linear patterns, and is suitable for multi-label classification datasets. Let the original dataset contain n samples and m features, each sample consists of a feature vector and a category label [15]. The feature vector belongs to the m-dimensional real space, and the category label belongs to a predefined category set. The dataset can be formally expressed as:

(1) $$D\;=\;\{(x_{i},y_{i})|x_{i}\in R^{m},y_{i}\in C,\;i\;=\;1,\;2,\;\ldots ,\;n\}$$

where $x_{i}$ is the feature vector of the i-th sample, $y_{i}$ is the corresponding category label, C is the category label set, and i is the total number of categories.

For the j-th feature $f_{j}$, its feature value vector is expressed as:

(2) $$f_{j}=\;(x_{1j},x_{2j},\ldots ,x_{\mathrm{nj}}{)}^{T}\in R^{n}$$

The corresponding label vector is:

(3) $$y=\;(y_{1},y_{2},\ldots ,y_{n}{)}^{T}\in C^{n}$$

Define the sorting mapping $\sigma_{j}$ to sort feature values in ascending order:

(4) $$x_{\sigma_{j}(1),j}\leq x_{\sigma_{j}(2),j}\leq \cdots \leq x_{\sigma_{j}(n),j}$$

According to the sorting mapping $\sigma_{j}$, the sorted label sequence $L_{j}$ is obtained:

(5) $$L_{j}=\;(y_{\sigma_{j}\left(1\right)},y_{\sigma_{j}\left(2\right)},\ldots ,y_{\sigma_{j}\left(n\right)})$$

In the label sequence $L_{j}$, the continuous pattern of category $c_{p}$ is defined as the maximum subsequence length of continuous occurrence of the category label.

Let $n_{p}$ be the total number of samples belonging to category $c_{p}$ in the dataset:

(6) $$n_{p}=|\{i|y_{i}=c_{p},i=1,2,\ldots ,n\}|,\sum_{p=1}^{l}n_{p}=n$$

Based on the above definition, the Maximum Pattern Recognition separability score $\rho_{j}$ of the j-th feature is defined as:

(7) $$\rho_{j}=\sqrt{\prod_{p=1}^{l}\frac{m_{p}^{j}}{n_{p}}},j=1,2,\ldots ,m$$

In Formula (7), $m_{p}^{j}$ represents the maximum number of consecutive occurrences of category $c_{p}$ in the label sequence sorted by the j-th feature, and $n_{p}$ represents the total number of samples of category $c_{p}$.

The MPR score has a clear value range, satisfying $\rho_{j}\in \;\left[0,\;1\right]$. When the score is equal to 1, it means that the feature can perfectly distinguish all categories, and all samples of each category appear continuously after sorting. When the score is close to 0, it means that the feature has poor separability, and labels of various categories are highly mixed in the sorted sequence. The higher the score, the stronger the separability of the feature, and the more suitable it is as a discriminative feature for classification tasks.

#### 3.2.3. MOPSO-ASFS

After the preliminary dimensionality reduction of high-dimensional features by the MPR filter, MOPSO-ASFS performs refined multi-objective optimization in the compressed feature space. With minimizing the feature selection rate and optimizing model performance as the core bi-objective, this algorithm regulates population selection pressure relying on the adaptive penalty mechanism of PBI decomposition [16], avoids local optima combined with the feature information-driven guide particle selection strategy, and finally outputs the Pareto optimal feature subset that meets the requirements of compact features, accurate classification, and stable results, which are specially adapted to the characteristics of high-dimensionality, small sample size, and strong noise of metabolomics data [17].

1. Fitness Evaluation

Fitness evaluation includes particle coding and bi-objective function design, which is the core link connecting particle search behavior with feature selection tasks.

The algorithm adopts the continuous particle swarm optimization framework and completes the conversion from continuous position to binary feature selection result through threshold mapping. Let the feature dimension after MPR dimensionality reduction be D, and the position of the i-th particle in the population is a D-dimensional continuous vector, with each dimension constrained to [0, 1]:

(8) $$x_{i}=\;(x_{i,1},x_{i,2},\ldots ,x_{i,D}),\;x_{i,j}\in \;[0,1],\;j=1,2,\ldots ,D,\;i=1,2,\ldots ,N\;$$

where N is the population size. The mapping from continuous value to binary coding is completed through a fixed threshold a = 0.6, and the decision rule is:

(9) $$s_{i,j}=\left\{\begin{array}{ll} 1, & x_{i,j}\geq a\\ 0, & x_{i,j}<a \end{array}\right.\;$$

$s_{i,j}$ = 1 means the j-th feature is selected, and $s_{i,j}$ = 0 means the feature is eliminated. This coding method takes into account the global search ability of continuous PSO and the binary decision requirement of feature selection.

In view of the triple requirements of compactness, accuracy, and stability for metabolomics feature selection, this algorithm designs two conflicting minimization objectives to replace the traditional optimization framework, which only focus on classification error rate and feature number. The first objective is the feature selection rate (denoted as $f_{1}$), defined as the ratio of the number of selected features to the total number of features, as shown in Formula (10):

(10) $$f_{1}=\frac{SD_{i}}{D},\;SD_{i}\;=\sum_{j=1}^{D}s_{i,j}$$

where $SD_{i}$ is the number of features selected by the i-th particle, and D is the total number of features after MPR dimensionality reduction.

The second objective is the joint indicator of accuracy and stability (denoted as $f_{2}$), defined as the harmonic mean ${\mathrm{ES}}_{\beta }$ of the error rate and Hamming distance of the selected feature subset. The calculation formula of ${\mathrm{ES}}_{\beta }$ score is:

(11) $${\mathrm{ES}}_{\beta }\;=\;(1+\beta^{2})\times \frac{E\times S}{\beta^{2}\times E+S}$$

In Formula (11), β is a key adjustment parameter used to flexibly assign the weight of accuracy and stability in the joint indicator: when β < 1, the indicator pays more attention to stability (the smaller β is, the greater the weight of stability in optimization); when β > 1, the indicator is more biased towards the optimization of accuracy (the larger β is, the higher the weight of accuracy); when β = 1, equal weight is assigned to accuracy and stability, and ${\mathrm{ES}}_{\beta }$ degenerates to the harmonic mean of E and S. In the MPR-MOPSO-ASFS multi-objective optimization algorithm, the ${\mathrm{ES}}_{\beta }$ score is used as the core optimization objective (minimization direction), and together with the feature selection rate forms a bi-objective optimization system. Finally, the Pareto optimum with low feature selection rate and low ${\mathrm{ES}}_{\beta }$ score is achieved, realizing the triple goals of compactness, accuracy, and stability of feature selection.

The error rate E is defined as 1 minus the classification accuracy (E = 1−Accuracy), and the smaller the value, the higher the model prediction accuracy; the stability S is calculated by the pairwise average Hamming distance of feature subsets after 10 Bootstrap resamplings. Hamming distance counts the number of features with inconsistent selection states between two independent runs, which directly reflects the reproducibility of metabolite screening results across different data subsets; the smaller the value, the stronger the stability of the feature selection results. The stability calculation method S is shown in Formula (12):

(12) $$S=\frac{2}{w(w-1)}\sum_{i=1}^{w-1}\sum_{j=1+1}^{w}\mathrm{HD}({\vec{x}}_{i},{\vec{x}}_{j})$$

In Formula (12), w is the number of Bootstrap resamplings, and $\mathrm{HD}({\vec{x}}_{i},{\vec{x}}_{j})$ is the Hamming distance of feature subsets after two Bootstrap resamplings.

2. Adaptive Penalty Archive Update Based on PBI Parameters

The Leader Archive (LA) is used to store high-quality non-dominated solutions, and its update quality determines the convergence and front uniformity of the algorithm. This algorithm adopts the adaptive penalty mechanism based on PBI decomposition to assign dynamic selection pressure to different weight vectors. The archive update algorithm is detailed in Algorithm 1 below.

The calculation formula of PBI aggregation function is:

(13) $$g(x|\lambda ,z^{*})=d_{1}+\theta \cdot d_{2}$$

(14) $$d_{1}=\frac{\|(F(x{)}^{T}-z^{*})\lambda \|}{\left\|\lambda \right\|},d_{2}=\|F(x)-(z^{*}+d_{1}\frac{\lambda }{\left\|\lambda \right\|})\|$$

where $z^{*}$ is the ideal point, λ is the weight vector, $d_{1}$ characterizes the convergence of the solution, $d_{2}$ characterizes the diversity of the solution, and θ is the penalty coefficient.

The adaptive penalty value update rule is:

(15) $$\theta_{i}(t)=1+\left({\mathrm{gen}}_{x}\cdot e^{-\sqrt{D}\cdot \mathrm{di}}\right)$$

(16) $$\mathrm{genx}=\theta_{\min}+(\theta_{\max}-\theta_{\min})\cdot \frac{\mathrm{gen}}{\mathrm{Maxgen}}$$

where di is the $d_{2}$ value of the optimal solution corresponding to the i-th weight vector, D is the dimension after MPR dimensionality reduction, $\theta_{\min}$ and $\theta_{\max}$ are the upper and lower limits of penalty values, gen is the current number of iterations, and Maxgen is the maximum number of iterations.

**Algorithm 1:** Update archive.
**Input:** LA (Leader Archive), LAS (maximum Leader Archive size)
**Output:** Updated LA, penalty values θ for each weight vector

1:for i = 1 to LAS2:   k ← Select the optimal particle from LA under $\theta_{i}$ according to Equations (13) and (14)3:   LA ← LA ∖ {k}4:   d_i ← Calculate the $d_{2}$ value of the ith weight vector according to Equation (14)5:   LA′ ← LA′ ∪ {k}6:end for7:Update all $\theta_{i}$ according to Equations (15) and (16)

3. Adaptive Guide Particle Selection Based on Feature Information

Stagnant particles are identified by the age threshold Ta, and adaptive guide particles are generated combined with feature frequency and opposition-based mutation to avoid premature convergence of the algorithm. The particle update procedure is presented in Algorithm 2 below.

The feature frequency calculation formula is:

(17) $$\mathrm{FR}=\sum_{k=1}^{\left|\mathrm{LA}\right|}s_{k}(t),\;s_{k}\left(t\right)\in \mathrm{LA}$$

where FR is the total number of times all features in the archive are selected, reflecting the global selection preference of features.

The guide particle update formula based on opposition-based mutation [18] is:

(18) $$x_{\mathrm{ij}}(t+1)=0.5\times \left(\mathrm{rand}{\left(\right)}_{\mathrm{ij}}\times (a_{\mathrm{ij}}+b_{\mathrm{ij}})-\mathrm{FR}+1\right)$$

where $a_{\mathrm{ij}}$ and $b_{\mathrm{ij}}$ are the upper and lower limits of particle position, and $\mathrm{rand}({)}_{\mathrm{ij}}$ is a random number from 0 to 1.

**Algorithm 2:** Update particles.
**Input:** P (population), T (neighbor size), LA (Leader Archive),
age (particle age), Ta (age threshold), θ (penalty values)
**Output:** New population P′, Updated particle age

1:for i = 1 to N do2:   if ${\mathrm{age}}_{i}<T\_a$ then3:      $B_{i}$ ← Find T neighboring weight vectors of $\omega_{i}$4:      ${\mathrm{gbest}}_{i}$ ← Select the optimal solution from the solutions corresponding to $B_{i}$ according to Equation (14)5:   else6:      FR ← Calculate feature frequency according to Equation (17)7:      ${\mathrm{gbest}}_{i}$ ← Reconstruct the leading particle according to Equation (18)8:   end if9:   Update the velocity and position of $P_{i}$ according to PSO velocity-position formula10:   ${\mathrm{age}}_{i}\leftarrow {\mathrm{age}}_{i}+1$11:   If $P_{i}$ dominates ${\mathrm{pbest}}_{i}$, update ${\mathrm{pbest}}_{i}$ and reset ${\mathrm{age}}_{i}=0$12:end for13:Update the ideal point $z^{*}$ based on P′ ∪ P


### 3.3. Computational Details

The proposed MPR-MOPSO-ASFS algorithm is implemented in Python 3.8+, with NumPy (1.24.3), pandas (2.2.3), scikit-learn (1.6.1), and Matplotlib (3.10.0) adopted for numerical computation, data preprocessing, model evaluation, and visualization. With a serial CPU architecture and no GPU dependency, the algorithm is compatible with mainstream operating systems. All experiments were conducted on a desktop workstation with an Intel Core i7-10700 CPU and 8 GB of RAM. In terms of time complexity, the first-stage MPR filter is O(d⋅nlogn)) (where d is the number of original features and n is the sample size), with negligible computational cost for pre-screening. The second-stage MOPSO-ASFS is $O(G\cdot N\cdot B\cdot n\cdot d^{\prime })$ (where G is the number of iterations, N is the population size, B is the number of Bootstrap resamplings, and $d^{\prime }$ is the number of reduced features), and its computational cost scales approximately linearly with core parameters.

### 3.4. Parameter Setting

To ensure the fairness of comparison among algorithms, all comparison algorithms are tested under unified experimental conditions. The experimental preprocessing process remains consistent: firstly, Z-score standardization is performed on the data to eliminate the influence of different feature dimensions; secondly, aiming at the possible class imbalance problem in metabolomics data, the SMOTE method is used to resample the training data to weaken the impact of class distribution bias on classification results [19].

In terms of classification performance evaluation, the K-nearest neighbor classifier with k = 5 is uniformly adopted in this paper [20]; in terms of stability evaluation, the average value of 1-Hamming distance between feature subsets is calculated through 10 Bootstrap resamplings to measure the consistency of feature selection results, and the larger the value, the stronger the stability. For multi-objective optimization results, Hypervolume (HV) and Inverted Generational Distance (IGD) indicators are used to evaluate the quality of the Pareto front [21].

The parameter settings of each comparison algorithm follow the recommended values in the original literature to ensure the objectivity of comparison results. For the MPR-MOPSO-ASFS method proposed in this paper, the parameter settings are as follows: population size N = 30, maximum number of iterations maxgen = 100, mapping threshold a = 0.6, particle stagnation threshold Ta = 10, and ${\mathrm{ES}}_{\beta }$ score parameter β = 1.

### 3.5. Evaluation Indicators

Hypervolume (HV) and Inverted Generational Distance (IGD) are classic indicators for evaluating the quality of the Pareto solution set of multi-objective optimization algorithms. HV can comprehensively reflect the convergence and distribution of the solution set. The larger the value, the wider the coverage of the target space, the more uniform the distribution, and the closer the algorithm output solution set is to the real Pareto front. IGD is mainly used to measure the approximation degree of the algorithm output solution set to the real Pareto front. The smaller the value, the better the convergence of the solution set. Specifically, HV measures the overall objective space covered by the solution set, reflecting the diversity and comprehensiveness of candidate biomarker combinations provided by the algorithm; IGD calculates the average distance from the obtained solutions to the reference optimal front, indicating how close the screened metabolite subsets are to the theoretical optimum. The combination of the two can comprehensively evaluate the performance of multi-objective optimization algorithms. The definition of HV is:

(19) $$\mathrm{HV}(\mathrm{PF})=\lambda \left(\underset{\vec{p}\in \mathrm{PF}}{⋃}\left\{\vec{x}\in R^{2}|\vec{p}≼\vec{x}≼{\vec{z}}_{\mathrm{ref}}\right\}\right)$$

where λ(·) is the Lebesgue measure in two-dimensional space, PF is the Pareto solution set output by the algorithm, ${\vec{z}}_{\mathrm{ref}}$ is the reference point (taken as the maximum value of the solution set target values of all comparison algorithms plus 0.1), and ≼ is the dominance relation (i.e., $\vec{p}≼\vec{x}$ means all target values of $\vec{p}$ are not greater than those of $\vec{x}$). The definition of IGD is:

(20) $$\mathrm{IGD}(\mathrm{PF},PF^{*})=\frac{1}{|PF^{*}|}\sum_{{\vec{x}}^{*}\in PF^{*}}\underset{\vec{x}\in \mathrm{PF}}{\min}{\left\|\vec{x}-{\vec{x}}^{*}\right\|}_{2}$$

where $PF^{*}$ represents the reference Pareto front obtained by merging and screening the solution sets of all comparison algorithms, $|PF^{*}|$ is the number of solutions in the reference front, and ‖ ‖_2_ is the Euclidean norm.

## 4. Results

### 4.1. Comparative Experiment

**Figure 2:**
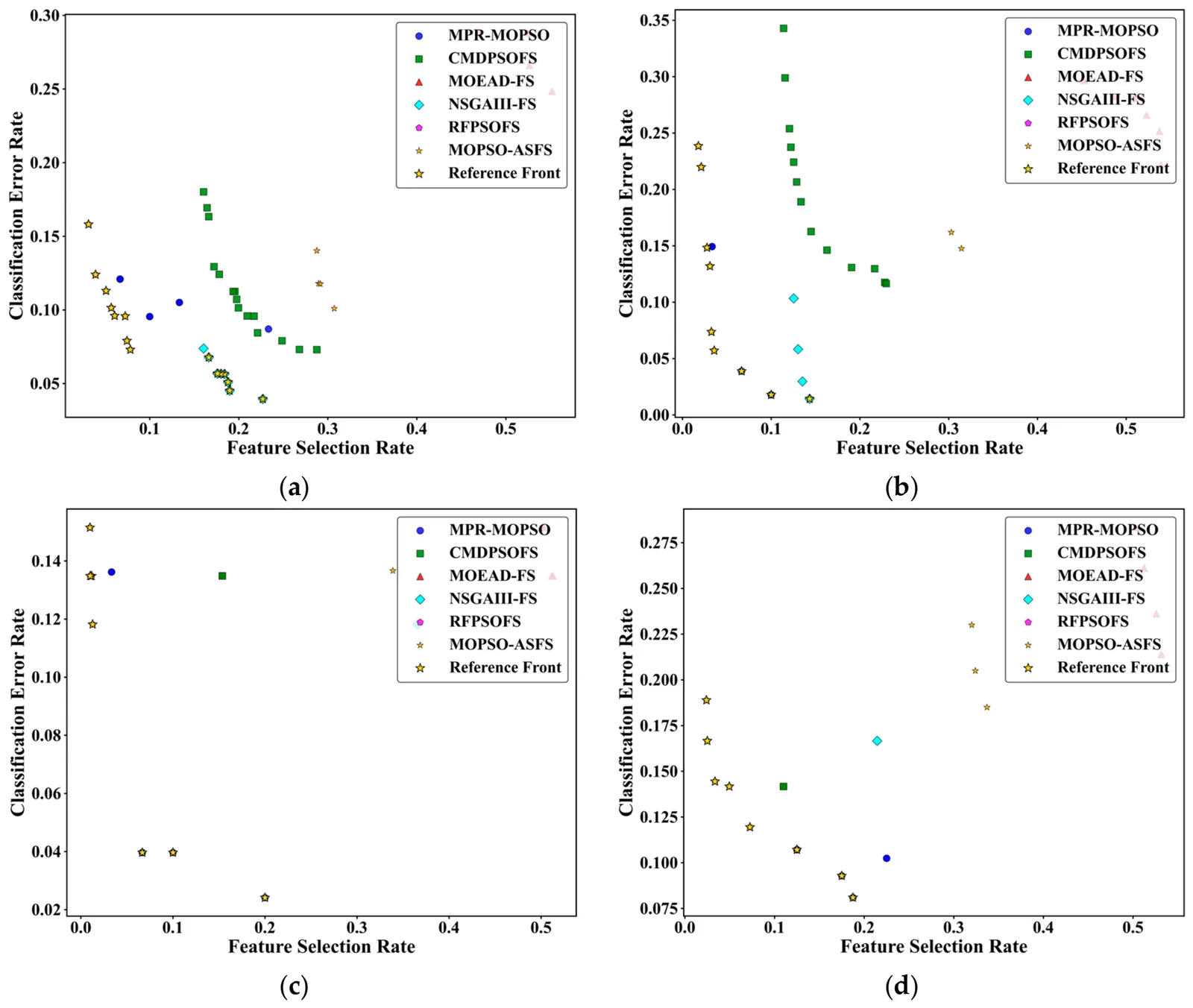

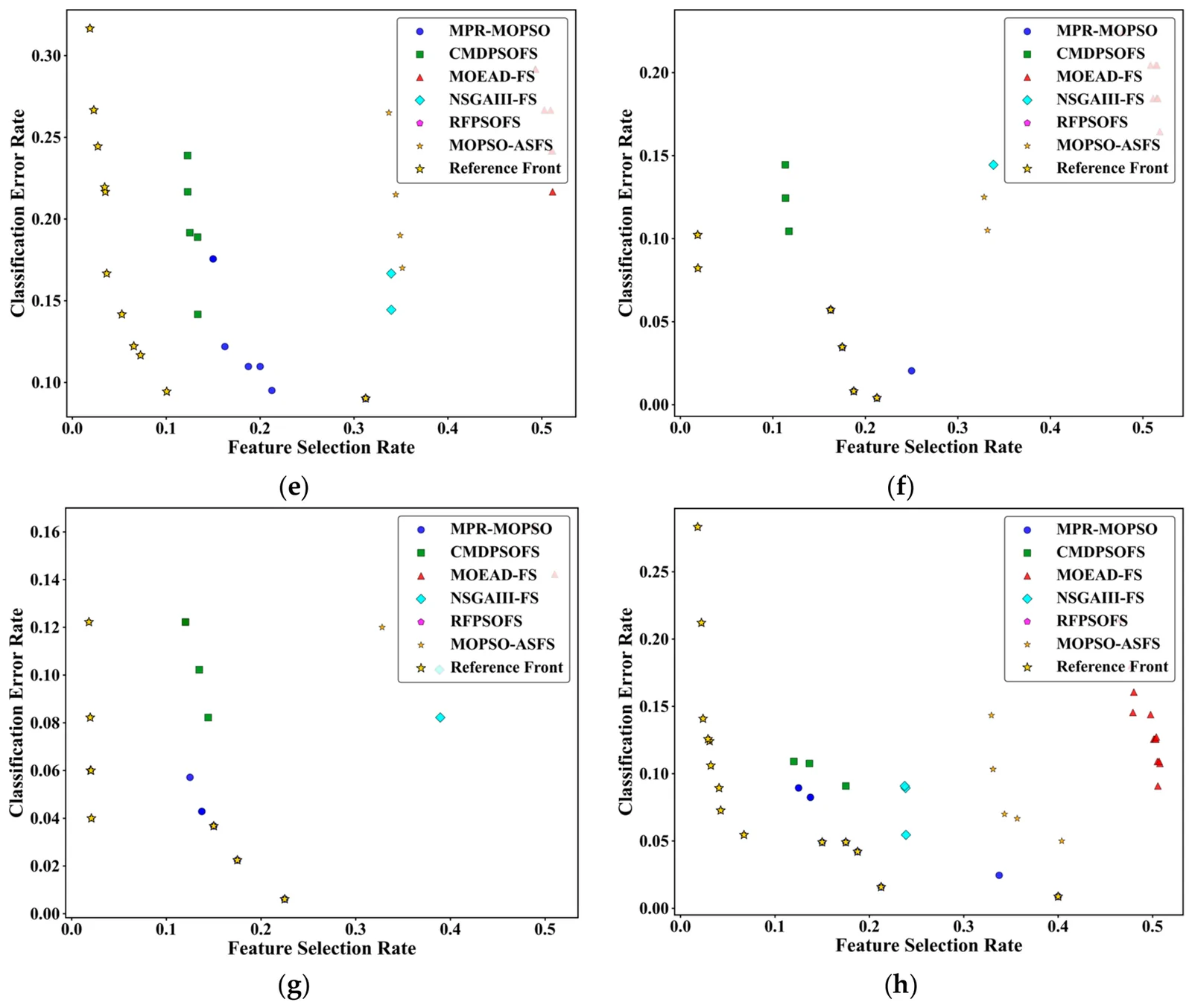
Comparative experiments on the ST000385, ST000419, EnSub, Colon, CNS, Leukemia, MLL, and SRBCT datasets from (**a**–**h**). The abscissa is the feature selection rate, and the ordinate is the classification error rate; each colored point represents the Pareto non-dominated solution set distribution of the MPR-MOPSO-ASFS, CMDPSOFS, MOEAD-FS, NSGAIII-FS, RFPSOFS, and MOPSO-ASFS algorithms, and the five-pointed star is the real reference Pareto front obtained by merging and screening the solution sets of all comparison algorithms.

**Figure 3:**
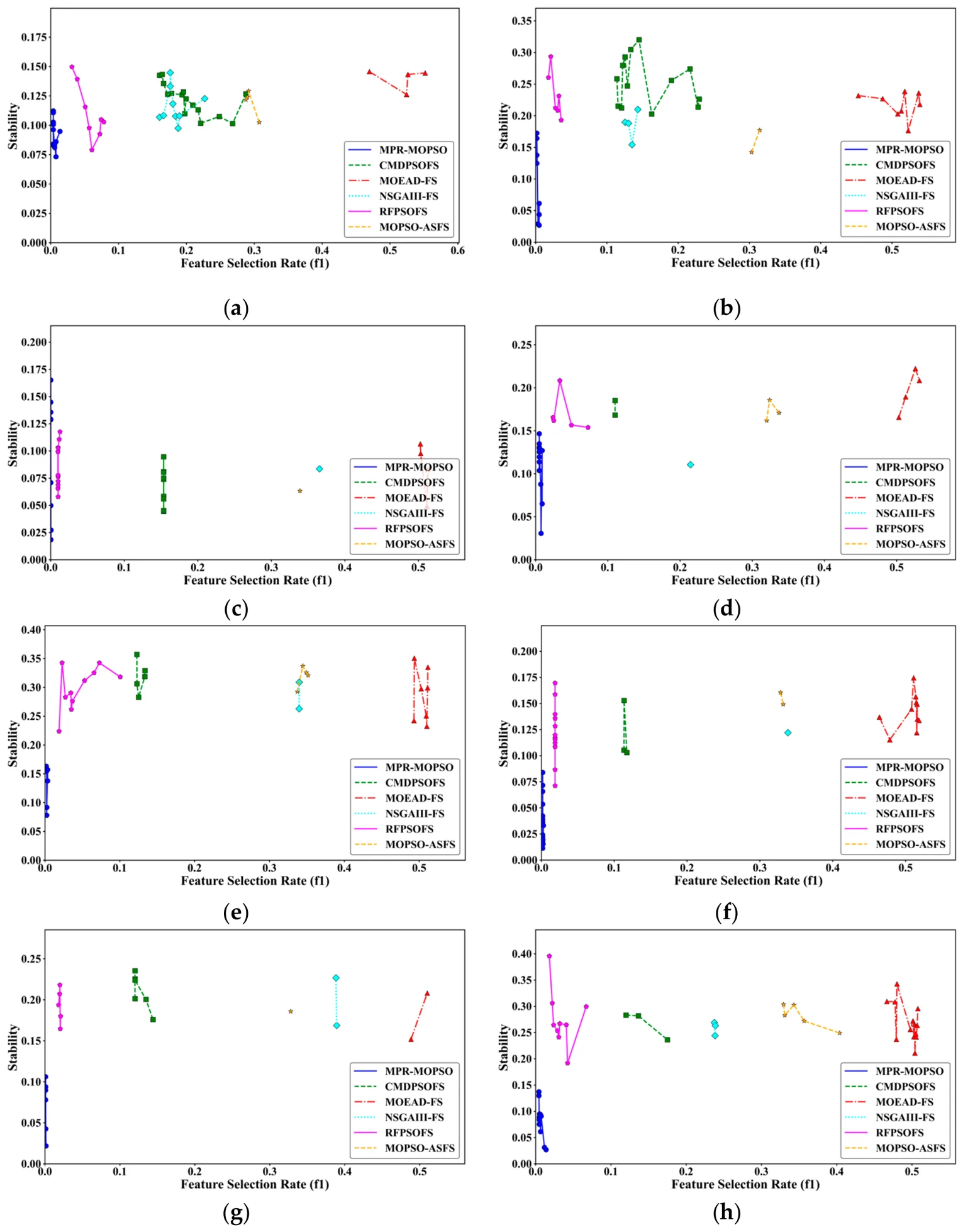
Comparative experiments on the ST000385, ST000419, EnSub, Colon, CNS, Leukemia, MLL, and SRBCT datasets from (**a**–**h**). The abscissa is the feature selection rate, and the ordinate is the stability represented by Hamming distance; each colored point represents the stability of non-dominated solutions of the MPR-MOPSO-ASFS, CMDPSOFS, MOEAD-FS, NSGAIII-FS, RFPSOFS, and MOPSO-ASFS algorithms.

The comparative experiment verifies the comprehensive advantages of the MPR-MOPSO-ASFS algorithm in feature compactness, classification accuracy, selection stability, and Pareto front quality on multiple datasets. The comparative experiment is randomly divided into a training set and an independent test set at a 7:3 ratio; all algorithms perform feature selection search within the training set, and classification accuracy is evaluated on the independent test set. The Pareto non-dominated solutions and stability of each algorithm are shown in Figure 2 and Figure 3, and the detailed data of each indicator are shown in Table 2.

From the perspective of Pareto front quality, the larger the HV value and the smaller the IGD value, the better the convergence and distribution of the solution set. On the metabolomics ST000385 dataset with obvious batch effects and high requirements for algorithm search adaptability [22], the HV value of the MPR-MOPSO-ASFS algorithm reaches 0.042, and the IGD value is only 0.044, which is only 53.0% of that of CMDPSOFS. This advantage is attributed to the MPR multi-disturbance adjustment module, which can dynamically adjust the search range and avoid falling into local optima, while algorithms with fixed neighborhood update strategies are not easily adapted to data feature fluctuations [23]. The HV value of the algorithm on other metabolomics datasets still ranks among the top, and the IGD value is lower; in the high-dimensional small-sample Leukemia dataset prone to the curse of dimensionality, its HV value is excellent, and the IGD value is only 80.2% of that of CMDPSOFS. The HV values of multiple high-dimensional small-sample datasets, such as MLL, SRBCT, CNS, and Colon, are also greatly improved compared with the comparison algorithms, and high-quality Pareto solution sets can be generated as a whole.

At the classification performance level, the test accuracy based on the K-nearest neighbor classifier with k = 5 can directly reflect the discrimination ability of the feature subset. In the EnSub metabolomics dataset with small sample category differences and high requirements for feature discrimination, the test accuracy of the MPR-MOPSO-ASFS algorithm reaches 92.3%, which is 3.8 percentage points higher than that of MOPSO-ASFS and CMDPSOFS, respectively. This is achieved by the ASFS adaptive sparsity mechanism accurately screening high-value features and filtering noise. On the high-dimensional small-sample Leukemia dataset prone to overfitting, its accuracy is 13.6 percentage points higher than that of NSGAIII-FS, and the accuracy of high-dimensional datasets such as CNS, Colon, MLL, and SRBCT is also better than that of the comparison algorithms.

The stability of feature selection is measured by the average Hamming distance of 10 Bootstrap resamplings, and the smaller the value, the stronger the stability. On the ST000385 metabolomics dataset, which is prone to feature selection result fluctuation affected by batch effects, the average stability of the MPR-MOPSO-ASFS algorithm is 0.092, which is only 65.7% of that of MOEAD-FS. The stability of other metabolomics datasets is also the best among all algorithms, with a decrease of nearly 50% compared with the comparison algorithms; on the high-dimensional small-sample Leukemia dataset where sample fluctuation has a significant impact on stability, its average stability is much lower than that of NSGAIII-FS and MOPSO-ASFS, with decreases of 69.7% and 76.1%, respectively. The stability of high-dimensional datasets, such as MLL, SRBCT, CNS, and Colon, is also reduced by more than 50% compared with the comparison algorithms.

In summary, the MPR-MOPSO-ASFS algorithm ranks among the top in four core indicators of HV, IGD, test accuracy, and feature stability in eight test datasets, and the overall comprehensive performance is better than the comparison algorithms.

### 4.2. Ablation Experiment

This experiment constructs two ablation variants around the core design of the algorithm, and verifies the independent role of each design by replacing the core mechanism, with the complete algorithm as the benchmark for comparison: the first variant (algo1) replaces the adaptive penalty value update rule in the algorithm with the penalty value strategy of MOPSO-ASFS presented in this paper to verify the impact of the adaptive penalty value update on the sparsity of feature selection and algorithm convergence efficiency; the second variant (algo2) replaces the optimization objective of the algorithm from ${\mathrm{ES}}_{\beta }$ with the traditional classification accuracy only, which removes the constraint of stability dimension to verify the improvement effect of the bi-objective harmonic mean design on the robustness of the algorithm output results; and the benchmark algorithm (algo3) is the complete MPR-MOPSO-ASFS algorithm, which retains the adaptive penalty value update rule and ${\mathrm{ES}}_{\beta }$ optimization objective as a comparison reference for the two variants. The ablation experiment splits the data into training and test sets at a 7:3 ratio with a random seed; all algorithm variants are run independently for 10 repetitions under unified parameters, with accuracy reported as the mean and standard deviation of the test set results.

The complete results of the ablation experiment are shown in Table 3. This study analyzes the mechanism of core design from two types of test objects: metabolomics datasets and public high-dimensional small-sample datasets, combined with accuracy and stability indicators.

In terms of metabolomics datasets, focusing on the EnSub dataset, the benchmark algorithm achieves the optimal classification accuracy (0.9308 ± 0.0705) here, and the accuracy of algo1 and algo2 decreases by 5.37% and 3.31%, respectively, which clearly verifies the key role of the adaptive penalty value update rule and the ${\mathrm{ES}}_{\beta }$ optimization objective in improving the classification performance of metabolomics data. On the ST000385 dataset, the accuracy of both variants is lower than that of the benchmark and the stability percentage is negative; the ST000419 dataset is a case where the variant accuracy is higher than the benchmark, but the stability is still inferior to the benchmark, confirming the guaranteed effect of bi-objective harmonic mean on stability. On the whole, the stability decline range of algo2 is generally larger than that of algo1.

In terms of high-dimensional small-sample datasets, taking the CNS dataset as an example, the benchmark algorithm achieves the optimal classification accuracy, and the accuracy of algo1 and algo2 decreases by 8.00% and 10.00%, respectively, which fully verifies the core role of the adaptive penalty value update rule in alleviating the curse of dimensionality—fixed penalty values cannot adapt to high-dimensional search requirements, and a single accuracy objective ignores robustness, both leading to a significant performance degradation. On ultra-high-dimensional datasets such as Leukemia and MLL, the accuracy and stability gaps between algo1 and the benchmark are extremely small because the mean penalty value can ensure basic sparsity; although the variants of the Colon and SRBCT datasets occasionally have accuracy improvement, the stability fluctuates significantly, showing randomness. In summary, the complete MPR-MOPSO-ASFS algorithm achieves balanced optimization of classification performance and stability through a dual-core design, avoiding the limitations of a single design.

### 4.3. Parameter Experiment

#### 4.3.1. Parameter mpr_top_k

**Figure 4:**
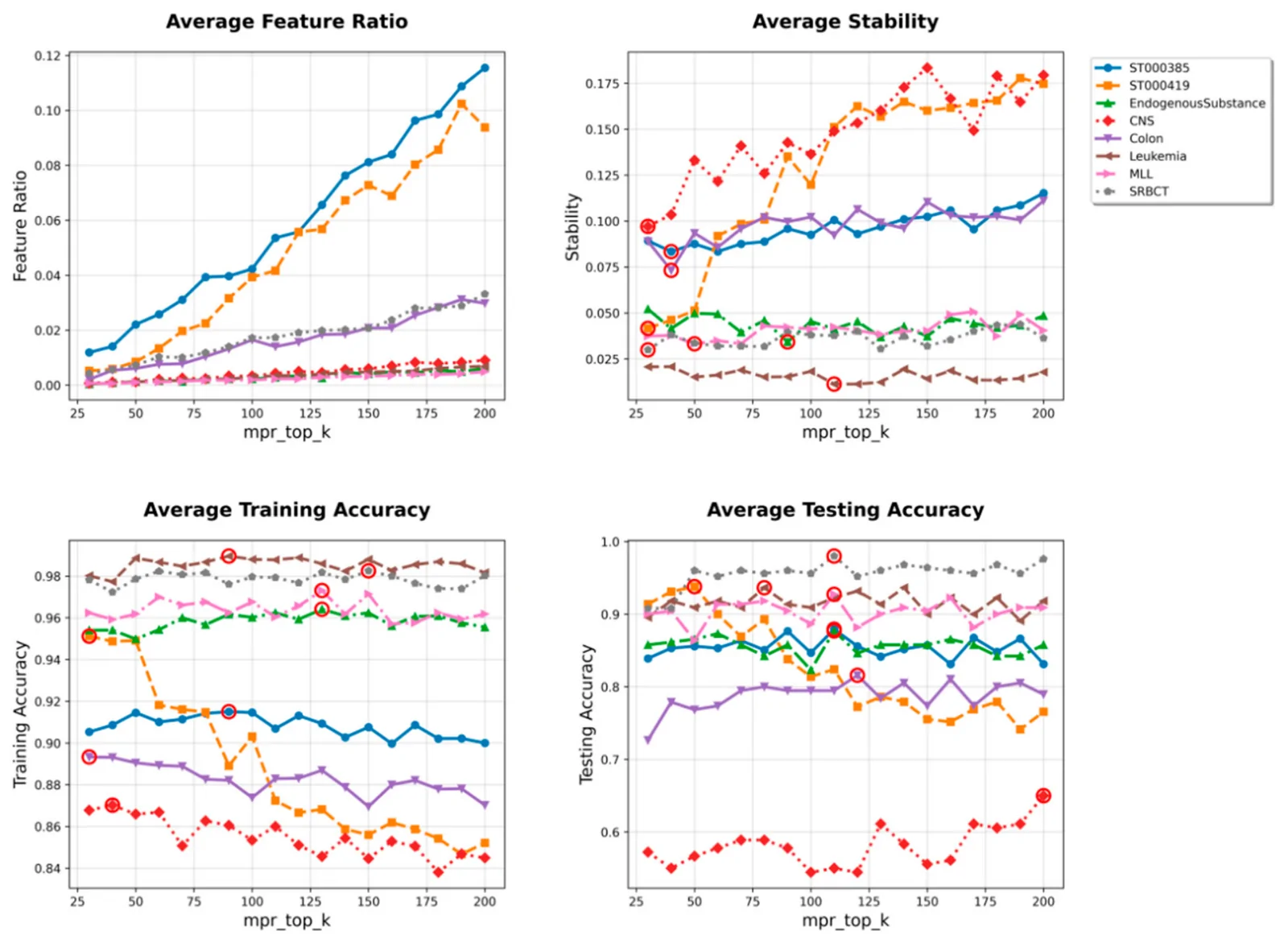
The figure contains four subgraphs, from top left to bottom right are the change trends of the average feature selection rate, average stability, average training accuracy, and average test accuracy with mpr_top_k; the abscissa is the value of mpr_top_k (30~200, step size 10), the ordinate is the corresponding performance index value, and different color curves represent 8 experimental datasets.

In this experiment, mpr_top_k is set as the only variable, and the test value range is 30~200 with a step size of 10, covering low, medium, and high candidate feature space scales; to avoid the random deviation of a single experiment, each parameter configuration runs independently 10 times, and the training set/test set is divided according to the 7:3 ratio with different random seeds each time to ensure the statistical reliability of the results. The experimental results are shown in Figure 4.

Based on the experimental results, the influence of mpr_top_k parameter change on algorithm performance shows differentiated rules under different types of datasets, and all reflect the non-linear correlation between parameters and performance as a whole. For metabolomics datasets with moderate feature dimensions, batch effects, and strong noise, focusing on ST000385 as an example, the performance impact of parameter changes shows a rule of first better then worse: the average feature selection rate of this dataset continues to rise as mpr_top_k increases from 30 to 200, the feature compactness gradually decreases, the average stability slightly decreases, and the average test set accuracy reaches the peak when mpr_top_k = 90. Too small parameters will lose high-value metabolic features, and too large parameters will introduce redundant noise features, both of which will reduce classification accuracy. The ST000419 dataset has a low original feature dimension; the test accuracy reaches the optimal when mpr_top_k = 50, and the accuracy decreases rapidly after the parameter increases. The feature selection rate of EnSub is always very low; the accuracy reaches the peak when mpr_top_k = 110, and the parameter change has a weak impact on its generalization ability. For public high-dimensional small-sample datasets with large feature dimension span and serious dimensional imbalance, taking the Leukemia ultra-high-dimensional dataset with 7129 dimensions as the core, its average feature selection rate is always maintained at a very low level; the average stability reaches the optimal when mpr_top_k = 110/120, and the average test set accuracy reaches the peak when mpr_top_k = 80. This parameter interval can achieve the best balance between retaining core gene features and filtering redundant noise. The Colon dataset with 2000 dimensions reaches the accuracy peak when mpr_top_k = 120, and too large parameters will increase the risk of overfitting; the CNS dataset with the same 7129 dimensions has more serious dimensional imbalance, and the accuracy continues to rise with the increase in parameters, requiring mpr_top_k = 200 to retain enough discriminative features.

Based on the experimental results of eight test datasets, the optimal value strategy of mpr_top_k can be clarified: the overall optimal value range of metabolomics datasets is 50~110, within which the algorithm can achieve the best balance between feature compactness, result stability, and model generalization ability; ultra-high-dimensional public datasets are recommended to be adjusted within the range of 80~200 according to dataset characteristics; datasets with concentrated feature distribution can take about 80, and datasets with scattered feature distribution and serious dimensional imbalance need to appropriately increase parameters; the optimal value of medium and high-dimensional public datasets is 120, taking into account feature compactness and generalization ability; and mpr_top_k = 80 is the general optimal parameter of the algorithm, which can achieve balanced performance of various indicators on most datasets, and is the preferred configuration when there is no customized demand. For specific datasets, fine-tuning can be carried out according to the principle of taking smaller top_k for low-feature-dimensional datasets and larger top_k for datasets with ultra-high-dimensional or serious dimensional imbalance.

#### 4.3.2. Parameter ${\mathrm{ES}}_{\beta }$

**Figure 5:**
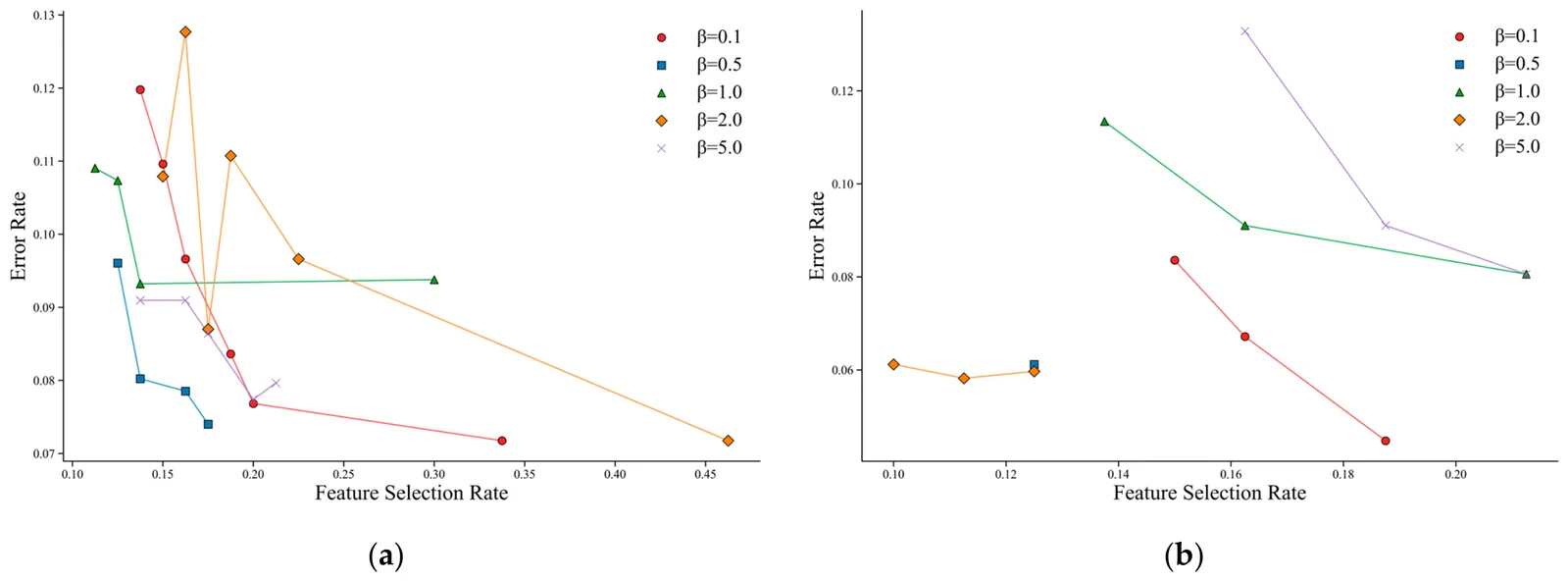

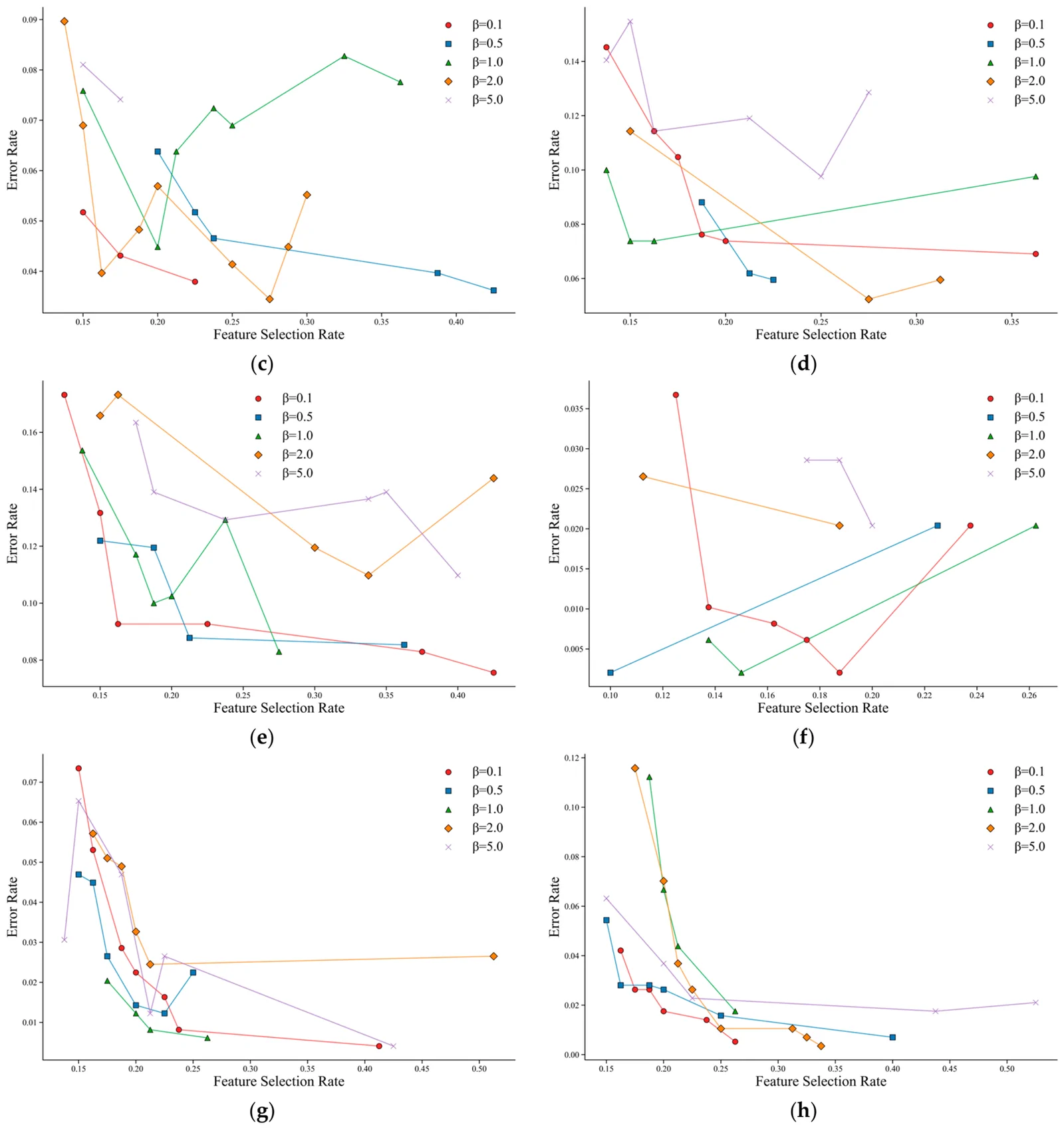
From (**a**–**h**), the figure shows the Pareto solution set distribution of the MPR-MOPSO-ASFS algorithm on the ST000385, ST000419, EnSub, Colon, CNS, Leukemia, MLL, and SRBCT datasets when β takes 0.1, 0.5, 1.0, 2.0, and 5.0 with different color curves. The abscissa is the feature selection rate, and the ordinate is the classification error rate.

To balance the compactness of feature selection, model classification performance, and algorithm result stability, the MPR-MOPSO-ASFS algorithm introduces the ${\mathrm{ES}}_{\beta }$ score as the core comprehensive evaluation indicator in the bi-objective optimization stage. The value of its parameter β directly determines the priority of the algorithm for classification error rate and result stability: when β < 1, the algorithm tends to optimize stability, adapting to application scenarios with high requirements for result robustness; when β > 1, the algorithm prioritizes improving classification accuracy, which is suitable for scenarios pursuing high classification performance; when β = 1, ${\mathrm{ES}}_{\beta }$ degenerates to arithmetic mean, achieving balanced optimization of the two indicators. Existing studies have not clarified the optimal value range of ${\mathrm{ES}}_{\beta }$ parameter β for metabolomics and high-dimensional small-sample data. To quantify the influence law of β on various algorithm performances and provide refined parameter configuration basis for algorithm practical application, this study designs an ${\mathrm{ES}}_{\beta }$ parameter sensitivity experiment, taking β as the only variable to carry out systematic tests. Five scenarios with β values of {0.1, 0.5, 1.0, 2.0, 5.0} are tested, covering the full interval of strong stability priority, balanced stability and accuracy, and strong accuracy priority. The comparison of Pareto fronts of the algorithm under different β values is shown in Figure 5, showing the characteristics of parameter preference differentiation and performance balance difference as a whole.

Based on the experimental results of eight test datasets, the optimal value strategy of ${\mathrm{ES}}_{\beta }$ parameter β can be clarified: for metabolomics application scenarios with extremely high requirements for result stability (such as disease marker screening, metabolite quantitative analysis verification) [24], it is recommended to select β = 0.1~0.5. The algorithm stability is optimal in this interval, although there is a slight loss of accuracy, it can be compensated by subsequent manual verification to ensure the repeatability of experimental results; for scenarios with high classification performance as the core goal and moderate requirements for stability (such as rapid disease diagnosis models), β = 2.0~5.0 is recommended. The algorithm classification accuracy reaches the peak in this interval, meeting the performance requirements of practical applications; for general scenarios pursuing comprehensive performance without clear priority bias, β = 1.0 is the general optimal parameter of the algorithm. This value achieves the best balance between stability and classification accuracy on all test datasets. The number of non-dominated solutions and the feature selection rate are in a reasonable range, no additional parameter fine-tuning is required for datasets, and it is the preferred configuration for the landing application of the MPR-MOPSO-ASFS algorithm.

### 4.4. Extended Experiment

The extended experiment replaces ${\mathrm{ES}}_{\beta }$ with the single accuracy of the usual optimization objective, and the relevant results are shown in Table 4.

From the experimental results of five algorithms, CMDPSOFS, MOEAD_FS, MOPSO_ASFS, NSGAIII_FS, and RFPSOFS, on eight datasets including Colon, SRBCT, MLL, CNS, Leukemia, ST000385, ST000419, and EnSub, the v2 version with the harmonic mean of accuracy and stability as the optimization objective generally shows a trend of performance improvement compared with the v1 version with the original optimization objective in most dataset and algorithm combinations. Among them, the v2 version of the RFPSOFS algorithm has the most prominent comprehensive performance, achieving the optimal value on multiple datasets such as Colon, SRBCT, MLL, and CNS. In particular, the v2 version of the RFPSOFS algorithm reaches 0.76 on the Colon dataset, which far exceeds the v1 version of the same algorithm and other algorithm versions, and it also leads in all results on the SRBCT dataset with 0.876. The overall performance of the CNS dataset is low, and the value of each algorithm version only floats in the range of 0.466~0.527; however, achieving high classification performance with the CNS dataset is difficult for all algorithms. The results of the v1 and v2 versions of each algorithm on the Leukemia dataset are similar with small fluctuations. Most v2 algorithms are better than v1 on the ST000385 and ST000419 datasets, and the performances of each of the v1 and v2 versions of the algorithms on the EnSub dataset are at a high level with little difference, with values concentrated in the range of 0.846~0.869.

## 5. Discussion

In this study, the comprehensive performance of the MPR-MOPSO-ASFS algorithm for feature selection on metabolomics and high-dimensional small-sample datasets is systematically verified through comparative experiments, ablation experiments, parameter sensitivity analysis, and extended experiments. Overall, the proposed algorithm outperforms existing mainstream multi-objective feature selection algorithms in four core indicators: Pareto front quality, classification accuracy, feature compactness, and selection stability. Its two core designs, namely the adaptive penalty value update rule and the ${\mathrm{ES}}_{\beta }$ bi-objective harmonic mean optimization objective, exhibit independent contributions and synergistic effects. The parameter configuration possesses favorable universality and scenario adaptability, and can provide refined parameter tuning strategies for different data characteristics.

Multiple experimental findings of this paper are highly consistent with the conclusions of existing domain research, which verifies the reliability of experimental observations. First, the ubiquitous batch effects and high-dimensional small-sample characteristics of metabolomics data are core factors restricting the performance of feature selection algorithms, which has been confirmed by multiple omics data mining studies [25,26]. In this paper, an obvious decline in selection stability of all comparison algorithms is observed on datasets with significant batch effects such as ST000385, which further confirms the negative impact of batch effects on the reproducibility of feature selection results. Second, traditional multi-objective particle swarm optimization algorithms with fixed neighborhood update strategies are prone to fall into local optima in high-dimensional non-convex search spaces, leading to imbalance between convergence and distribution of the Pareto front [27]. The HV and IGD performance of baseline algorithms such as CMDPSOFS in our comparative experiments also conforms to this universal rule. Third, the overfitting problem caused by the curse of dimensionality in high-dimensional small-sample scenarios is a common challenge for all feature selection algorithms [28]. The generally decreased classification accuracy of each comparison algorithm on ultra-high-dimensional datasets such as Leukemia and CNS in this study is fully consistent with existing research conclusions.

In terms of objective function design, the vast majority of existing multi-objective feature selection algorithms take “classification error rate + feature subset size” as dual optimization objectives [11,29], which only focus on classification performance and feature compactness in a single run without incorporating selection stability across multiple runs into the optimization framework. To address this research gap, this paper proposes the ${\mathrm{ES}}_{\beta }$ harmonic mean optimization objective, which weights and integrates feature selection stability and classification performance to directly guide the algorithm to search for feature subsets with both discriminability and robustness. The extended experimental results show that this objective function can be transferred to various mainstream multi-objective feature selection algorithms, such as CMDPSOFS and RFPSOFS, and achieve performance improvement. It breaks through the objective dimension limitation of traditional multi-objective feature selection and is one of the core innovations of this paper.

In terms of search strategy optimization, most existing multi-objective particle swarm feature selection algorithms adopt fixed penalty values and fixed neighborhood update mechanisms [30], which cannot adaptively match the complex distribution of high-dimensional data. The adaptive penalty value update rule designed in this paper can dynamically adjust the sparsity constraint intensity according to the particle iteration state, and cooperates with the MPR multi-disturbance adjustment module to dynamically compress the search space, effectively alleviating the defect that fixed strategies are prone to fall into local optima. Ablation experiments show that this design has a particularly significant effect on improving classification performance in ultra-high-dimensional datasets with more than 7000 features, and is more adaptable to the curse of dimensionality in high-dimensional small-sample scenarios than fixed penalty value strategies.

## 6. Conclusions

Aiming at the characteristics of high-dimensionality, small sample size, strong redundancy, and high noise in metabolomics data, as well as addressing the problem of the existing multi-objective feature selection algorithms ignoring stability optimization and having insufficient adaptability to high-dimensional data, this paper proposes the MPR-MOPSO-ASFS algorithm. Taking minimizing the feature selection rate $f_{1}$ and the harmonic mean of accuracy and stability $f_{2}$ as bi-objectives, this method performs pre-dimensionality reduction through the MPR filter and optimizes the particle search process combined with the ASFS mechanism, in order to realize the collaborative optimization of feature selection compactness, classification performance, and result stability. The experimental results on three metabolomics datasets and five public high-dimensional small-sample datasets show that the MPR-MOPSO-ASFS algorithm is overall superior to the comparison algorithms in terms of Pareto front quality, classification accuracy, and feature selection stability, indicating that the proposed method can be well adapted to the feature selection tasks of metabolomics and high-dimensional small-sample data. Relevant ablation experiments and parameter experiments further verify the effectiveness of the bi-objective optimization design and each core mechanism.

Nevertheless, this study still has certain limitations. For example, the scale of experimental data is still limited, and it has not been further verified in large-scale multi-center and multi-batch metabolomics data; the feature evaluation process is mainly based on statistical information, and biological prior knowledge, such as metabolic pathways and metabolite interactions, has not been fully integrated; in addition, the algorithm is currently mainly oriented to supervised learning scenarios, and there is still room for further improvement in computational efficiency and application scope. Future research will focus on the integration of biological prior knowledge, parallel optimization, and the expansion of unsupervised and semi-supervised scenarios to further improve the interpretability, efficiency, and generalization ability of the method.

## Figures and Tables

**Table 1:** Datasets.

Dataset Name	Features	Samples	Classes
EnSub ^1^	10,283	54	3
ST000385	511	181	2
ST000419	614	80	2
Colon	2000	62	2
CNS	7129	60	2
Leukemia	7129	72	2
MLL	12,582	72	3
SRBCT	2308	83	4

^1^ The Shenfu Injection experimental dataset is constrained by the data disclosure policy of the funding project, and thus cannot be publicly released for the time being.

**Table 2:** Comparative experiment results.

Dataset	Algorithm	HV	IGD	Test_Acc	Avg_Sta
ST000385	MPR-MOPSO-ASFS	0.042	0.044	0.857	0.092
	CMDPSOFS	0.027	0.083	0.883	0.122
	MOEAD-FS	0.000	0.410	0.792	0.140
	NSGAIII-FS	0.035	0.054	0.857	0.116
	RFPSOFS	0.053	0.055	0.844	0.110
	MOPSO-ASFS	0.006	0.178	0.870	0.119
ST000419	MPR-MOPSO-ASFS	0.062	0.028	0.862	0.095
	CMDPSOFS	0.023	0.130	0.724	0.254
	MOEAD-FS	0.000	0.452	0.759	0.217
	NSGAIII-FS	0.037	0.082	0.862	0.185
	RFPSOFS	0.061	0.034	0.759	0.233
	MOPSO-ASFS	0.000	0.267	0.690	0.160
EnSub	MPR-MOPSO-ASFS	0.055	0.018	0.923	0.097
	CMDPSOFS	0.017	0.138	0.885	0.069
	MOEAD-FS	0.000	0.471	0.846	0.084
	NSGAIII-FS	0.000	0.333	0.885	0.084
	RFPSOFS	0.039	0.034	0.846	0.086
	MOPSO-ASFS	0.000	0.309	0.885	0.063
CNS	MPR-MOPSO-ASFS	0.083	0.101	0.444	0.137
	CMDPSOFS	0.079	0.104	0.556	0.319
	MOEAD-FS	0.000	0.424	0.500	0.287
	NSGAIII-FS	0.020	0.260	0.556	0.286
	RFPSOFS	0.121	0.035	0.611	0.298
	MOPSO-ASFS	0.018	0.265	0.389	0.319
Colon	MPR-MOPSO-ASFS	0.032	0.032	0.789	0.110
	CMDPSOFS	0.026	0.057	0.632	0.177
	MOEAD-FS	0.000	0.432	0.737	0.196
	NSGAIII-FS	0.009	0.128	0.632	0.111
	RFPSOFS	0.043	0.044	0.632	0.169
	MOPSO-ASFS	0.000	0.239	0.684	0.173
Leukemia	MPR-MOPSO-ASFS	0.029	0.069	0.909	0.037
	CMDPSOFS	0.019	0.086	0.864	0.120
	MOEAD-FS	0.000	0.400	0.818	0.142
	NSGAIII-FS	0.000	0.255	0.773	0.122
	RFPSOFS	0.035	0.083	0.818	0.122
	MOPSO-ASFS	0.000	0.237	0.773	0.155
MLL	MPR-MOPSO-ASFS	0.041	0.069	0.773	0.077
	CMDPSOFS	0.028	0.100	0.636	0.212
	MOEAD-FS	0.000	0.425	0.773	0.180
	NSGAIII-FS	0.000	0.312	0.727	0.198
	RFPSOFS	0.056	0.061	0.864	0.193
	MOPSO-ASFS	0.000	0.262	0.773	0.186
SRBCT	MPR-MOPSO-ASFS	0.135	0.073	0.880	0.083
	CMDPSOFS	0.110	0.102	0.800	0.267
	MOEAD-FS	0.007	0.385	0.840	0.269
	NSGAIII-FS	0.086	0.169	0.920	0.259
	RFPSOFS	0.156	0.057	0.880	0.276
	MOPSO-ASFS	0.055	0.243	0.880	0.282

**Table 3:** Ablation experiment results.

Dataset	Algorithm	Accuracy	Stability	Accuracy Gap (%)	Stability Gap (%)
ST000385	algo1	0.8532 ± 0.0247	0.8162	−1.65	−2.85
	algo2	0.8610 ± 0.0175	0.8101	−0.75	−3.57
	algo3	0.8675 ± 0.0265	0.8401	—	—
ST000419	algo1	0.8379 ± 0.0618	0.8312	3.85	−0.91
	algo2	0.8379 ± 0.0409	0.8049	3.85	−4.04
	algo3	0.8069 ± 0.0493	0.8388	—	—
EnSub	algo1	0.8808 ± 0.0698	0.8711	−5.37	4.79
	algo2	0.9000 ± 0.0255	0.8208	−3.31	−1.26
	algo3	0.9308 ± 0.0705	0.8313	—	—
CNS	algo1	0.5111 ± 0.0544	0.8011	−8.00	−3.67
	algo2	0.5000 ± 0.0430	0.8226	−10.00	−1.09
	algo3	0.5556 ± 0.0609	0.8316	—	—
Leukemia	algo1	0.9318 ± 0.0305	0.8765	−0.49	−0.02
	algo2	0.9000 ± 0.0727	0.8756	−3.88	−0.12
	algo3	0.9364 ± 0.0464	0.8767	—	—
Colon	algo1	0.7421 ± 0.0598	0.8458	−0.70	−1.52
	algo2	0.7737 ± 0.0668	0.8130	3.52	−5.33
	algo3	0.7474 ± 0.0516	0.8588	—	—
MLL	algo1	0.8045 ± 0.0540	0.8231	−0.56	0.54
	algo2	0.7818 ± 0.0833	0.8438	−3.37	3.07
	algo3	0.8091 ± 0.0490	0.8187	—	—
SRBCT	algo1	0.9360 ± 0.0196	0.7827	−3.31	1.08
	algo2	0.9160 ± 0.0377	0.8523	−5.37	10.08
	algo3	0.9680 ± 0.0392	0.7743	—	—

**Table 4:** Extended experiment results.

	CMDPSOFS		MOEAD_FS		MOPSO_ASFS		NSGAIII_FS		RFPSOFS	
	v1	v2	v1	v2	v1	v2	v1	v2	v1	v2
Colon	0.678	0.689	0.684	0.684	0.673	0.694	0.673	0.673	0.695	0.76
SRBCT	0.84	0.864	0.832	0.844	0.824	0.824	0.852	0.852	0.848	0.876
MLL	0.709	0.704	0.704	0.722	0.704	0.713	0.722	0.727	0.745	0.75
CNS	0.483	0.505	0.505	0.5	0.494	0.483	0.466	0.483	0.511	0.527
Leukemia	0.795	0.804	0.772	0.781	0.79	0.763	0.777	0.781	0.795	0.786
ST000385	0.826	0.839	0.809	0.819	0.824	0.850	0.832	0.845	0.836	0.846
ST000419	0.78	0.779	0.710	0.737	0.751	0.755	0.786	0.765	0.747	0.816
EnSub	0.861	0.861	0.853	0.857	0.865	0.869	0.857	0.861	0.846	0.846

## Data Availability

The original contributions and most supporting datasets presented in this study are included in the Supplementary Materials. One dataset (EnSub) used in this study is subject to third-party data use agreement restrictions and is not publicly available. Reasonable requests for access to the restricted dataset may be directed to the corresponding author.

## Supplementary Materials

The following supporting information can be downloaded at: https://www.mdpi.com/article/10.3390/metabo16080581/s1, Table S1: CNS; Table S2: Colon; Table S3: Leukemia; Table S4: MLL; Table S5: SRBCT; Table S6: ST000385; Table S7: ST000419; Table S8: Confusion matrix on the test set (MPR-MOPSO-ASFS). Dataset Notes introduces the datasets from Table S1 to Table S5, and MPR-MOPSO-ASFS.py provides the source code of the MPR-MOPSO-ASFS algorithm proposed in this study.
