# Supplementary material for: MPR-MOPSO-ASFS: A Stable Multi-Objective Feature Selection Algorithm for Metabolomics
Source: Metabolites. 2026 Aug 17;16(8):581. doi: 10.3390/metabo16080581 (PMC13515157; doi:10.3390/metabo16080581)
Supplement: Supplementary file 1 [file metabolites-16-00581-s001.zip › Dataset Notes.pdf]

1. Colon

The Colon dataset is derived from the classic study published by Alon et al. in Proceedings of the National Academy of Sciences of the United States of America in 1999, which belongs to research on transcriptome differential analysis and binary classification diagnosis between tumor and normal tissues[1]. This study aims to dissect genome-wide transcription patterns of colon adenocarcinoma and normal colonic mucosa via gene expression profiles, reveal the broad-spectrum molecular characteristics of tumorigenesis, and verify the feasibility of applying gene expression profiles to distinguish benign and malignant tissues. A total of 62 human colon tissue samples are included in this dataset, consisting of 40 colon adenocarcinoma tissues and 22 normal colonic epithelial tissues, with partial samples being paired tumor and paracancerous normal tissues from the same patients.

2. CNS

The CNS dataset originates from the central nervous system tumor study published by Pomeroy et al. in Nature in 2002, which is a multi-objective study covering molecular classification, pathogenesis elucidation and prognosis prediction of brain tumors[2]. This study aims to analyze the molecular heterogeneity of pediatric central nervous system embryonal tumors, define the molecular boundary between medulloblastoma and other embryonal tumors, validate the developmental biology hypothesis that medulloblastoma arises from cerebellar granule cells, and establish a patient survival prognosis prediction model based on transcriptomic features. This dataset contains 99 samples in total, including medulloblastoma, primitive neuroectodermal tumor, atypical teratoid/rhabdoid tumor, malignant glioma and normal brain tissue controls. Among them, 60 medulloblastoma samples are matched with complete clinical prognostic information and can be used for survival analysis and prognostic modeling.

3. Leukemia

The Leukemia dataset is derived from the landmark study published by Golub et al. in Science in 1999, which falls into the field of molecular classification and class prediction for hematological malignancies[3]. This study proposed a general cancer classification framework based on genome-wide expression profiles, aiming to break through the limitations of traditional morphological diagnosis, achieve unbiased molecular classification of acute lymphoblastic leukemia and acute myeloid leukemia, and establish a generalizable methodological system for tumor class prediction. A total of 72 leukemia samples from bone marrow or peripheral blood are included, divided into an independent training set and a test set. The training set comprises 38 samples (27 acute lymphoblastic leukemia cases and 11 acute myeloid leukemia cases), and the test set comprises 34 samples (20 acute lymphoblastic leukemia cases and 14 acute myeloid leukemia cases), covering both pediatric and adult patients.

4. MLL

The MLL dataset is derived from the leukemia subtype study published by Armstrong et al. in Nature Genetics in 2002, which focuses on molecular identification and pathogenesis of high-risk leukemia subtypes[4]. This study aims to characterize the unique transcriptomic signature of acute leukemia harboring mixed-lineage leukemia (MLL) gene rearrangement, demonstrate at the genomic level that this leukemia entity is a distinct disease subtype independent of conventional acute lymphoblastic leukemia and acute myeloid leukemia, and provide a theoretical basis for molecular diagnosis, risk stratification and targeted therapy of this high-risk leukemia. This dataset contains 72 leukemia samples from bone marrow or peripheral blood of newly diagnosed patients, divided into three groups: 24 cases of common B-cell acute lymphoblastic leukemia, 20 cases of leukemia with MLL gene rearrangement, and 28 cases of acute myeloid leukemia.

5. SRBCT

The SRBCT dataset originates from the pediatric oncology study published by Khan et al. in Nature Medicine in 2001, which belongs to research on differential diagnosis of multiple pediatric solid tumor subtypes[5]. Focusing on four types of pediatric small round blue cell tumors that are morphologically highly similar and indistinguishable under routine pathological examination, this study constructed an artificial neural network classification model based on gene expression profiles, aiming to achieve accurate identification and automated diagnosis of tumor subtypes and address clinical pain points in pediatric tumor pathological diagnosis. A total of 83 samples (including tumor biopsy tissues and corresponding cell lines) are included, divided into a 63-sample training set and a 20-sample independent test set, covering four tumor subtypes: Ewing sarcoma, neuroblastoma, Burkitt lymphoma and rhabdomyosarcoma.

References

1. Alon, U.; Barkai, N.; Notterman, D.A.; et al. Broad patterns of gene expression revealed by clustering analysis of tumor and normal colon tissues probed by oligonucleotide arrays. Proc. Natl. Acad. Sci. U. S. A. 1999, 96, 6745–6750. https://doi.org/10.1073/pnas.96.12.6745
2. Pomeroy, S.L.; Tamayo, P.; Gaasenbeek, M.; et al. Prediction of central nervous system embryonal tumour outcome based on gene expression. Nature 2002, 415, 436–442. https://doi.org/10.1038/415436a
3. Golub, T.R.; Slonim, D.K.; Tamayo, P.; et al. Molecular classification of cancer: class discovery and class prediction by gene expression monitoring. Science 1999, 286, 531–537. https://doi.org/10.1126/science.286.5439.531
4. Armstrong, S.A.; Staunton, J.E.; Silverman, L.B.; et al. MLL translocations specify a distinct gene expression profile that distinguishes a unique leukemia. Nat. Genet. 2002, 30, 41–47. https://doi.org/10.1038/ng813
5. Khan, J.; Wei, J.S.; Ringnér, M.; et al. Classification and diagnostic prediction of cancers using gene expression profiling and artificial neural networks. Nat. Med. 2001, 7, 673–679. https://doi.org/10.1038/89044
